# Validation of a Novel Genomic Biomarker of Mesenchymal Stem Cell Scalability and Implications of Genotype Status on Cellular Senescence Phenotypes

**DOI:** 10.21203/rs.3.rs-7115326/v1

**Published:** 2025-07-31

**Authors:** I Kade Karisma Gita Ardana, Vitali Maldonado, C. Lowry Barnes, Rebekah Margaret Samsonraj

**Affiliations:** University of Arkansas; University of Arkansas; University of Arkansas for Medical Sciences; University of Arkansas

**Keywords:** Mesenchymal stem cells, Senescence, Biomarker, GSTT1 polymorphism, Cell expansion, Cell therapies

## Abstract

Ex vivo expansion impairs the regenerative potential of bone marrow-derived mesenchymal stem cells (BM-MSCs), primarily by inducing cellular senescence. Interestingly, populations of BM-MSCs that exhibit resistance to senescence even after prolonged expansion have been reported. However, a reliable strategy to identify these populations is still underway. Previously, the GSTT1 gene has been identified as a biomarker for BM-MSC scalability but its effects on BM-MSC senescence have not yet been studied. Here, we investigate the role of GSTT1 genotype in BM-MSC senescence. First, we identified the GSTT1 genotype (either homozygous positive, heterozygous, or homozygous negative) of nine BM-MSC groups. Then, we performed long-term in vitro culture and exposed cells to irradiation as senescence models. After that, their proliferative potential, their SASP, and the expression of key genes were investigated. The results show that GSTT1 null BM-MSCs have a higher proliferative potential and exhibit fewer senescent cells in culture when compared to the other genotypes. Additionally, these cells exhibit a lower expression of p21 Waf1, p14ARF, IL-6, PDXN, and 53BP1 and a higher expression of TWIST1 and ACTA2 genes, especially at low passages. A GSTT1 null genotype can serve as a potential biomarker to identify BM-MSC populations with higher resistance to senesce.

## INTRODUCTION

Although bone marrow-derived mesenchymal stem cells (BM-MSCs) have great potential for treating multiple diseases [1–3], the lack of consistent and/or effective treatment outcomes poses a significant challenge for their clinical translation [4–7]. Multiple factors can play a role in hindering the therapeutic potential of BM-MSCs including the process of ex vivo expansion, which, although necessary for obtaining a clinically relevant number of cells [8], pushes cells towards senescence - a state or irreversible growth arrest - altering their phenotype and lowering their therapeutic potential [9–12]. Furthermore, it has been observed that donor-donor heterogeneity plays a critical role in dictating the potency and the onset of senescence in BM-MSCs [13–15], suggesting the existence of intrinsic inter donor differences that provide resistance to senescence. However, to date, these donor-donor differences are poorly understood, limiting the possibility of utilizing BM-MSC screening methods to improve BM-MSC therapies.

The GSTT1 gene, a member of the glutathione S-transferase superfamily which is not present in about 14–30% of the population’s genome [16], has been studied in the context of disease development and progression. For example, individuals with null GSTT1 gene seem to be at higher risk of developing cancer, Parkinson’s disease, and have a higher mortality rate due to COVID-19 [17–20]. This increased vulnerability may be associated with the primary function of the enzyme coded by this gene: the detoxification of various endogenous and exogenous substances including oxidative stress products, major players in cellular damage and senescence [21, 22]. Moreover, Sathiyanathan et al. reported that BM-MSCs from individuals with GSTT1 null genotypes grow faster and possess longer telomeres than those from individuals with the homozygous positive genotypes [23] incentivizing more research on these somewhat contradicting but important results.

This study aims to investigate the effect of the GSTT1 polymorphism (homozygous positive, heterozygous, or homozygous negative) on BM-MSC growth and senescence. First, the genomic status of nine BM-MSC groups was determined.. Then, cell senescence was induced through both in vitro irradiation and continuous cell passaging. Finally, BM-MSC proliferative capacity, telomere length, senescence-associated secretory phenotype (SASP), and gene expression profiles were assessed at different time points to determine whether GSTT1 genotype status has any relationship with the onset of cell senescence.

## RESULTS

### 1. Genotyping allowed BM-MSCs grouping into three categories determined by their GSTT1 status

**Figure 1:**
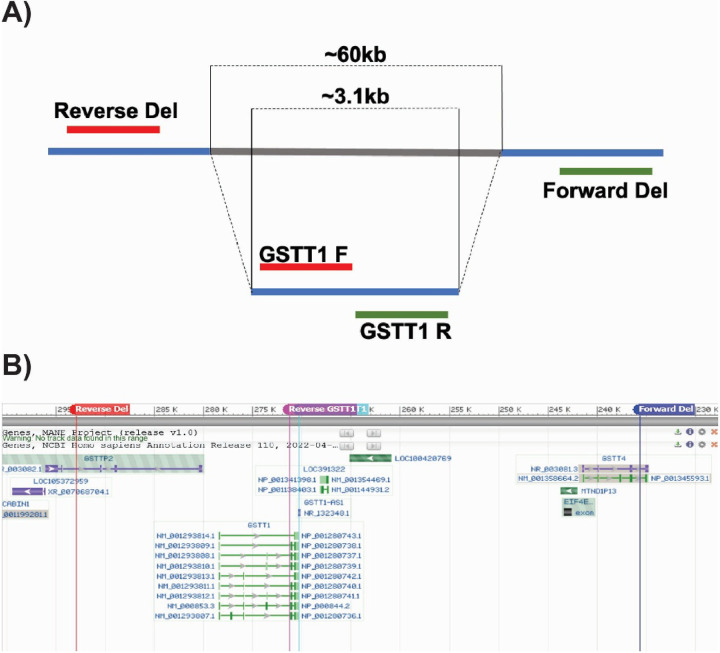
Location of the primers used for genotyping through multiplexing PCR. **A)** When present, the GSTT1 gene will be amplified using the GSTT1 F and GSTT1 R primers generating a 3.1kb product. The GSTT1 deletion will only be amplified when the GSTT1 gene is missing since the product of Reverse Del and Forward Del is too big to be amplified when present (60kb). **B)** Genome location of the GSTT1 gene as well as the primer amplification site for the GSTT1 gene and the deletion of the GSTT1 gene.

**Figure 2:**
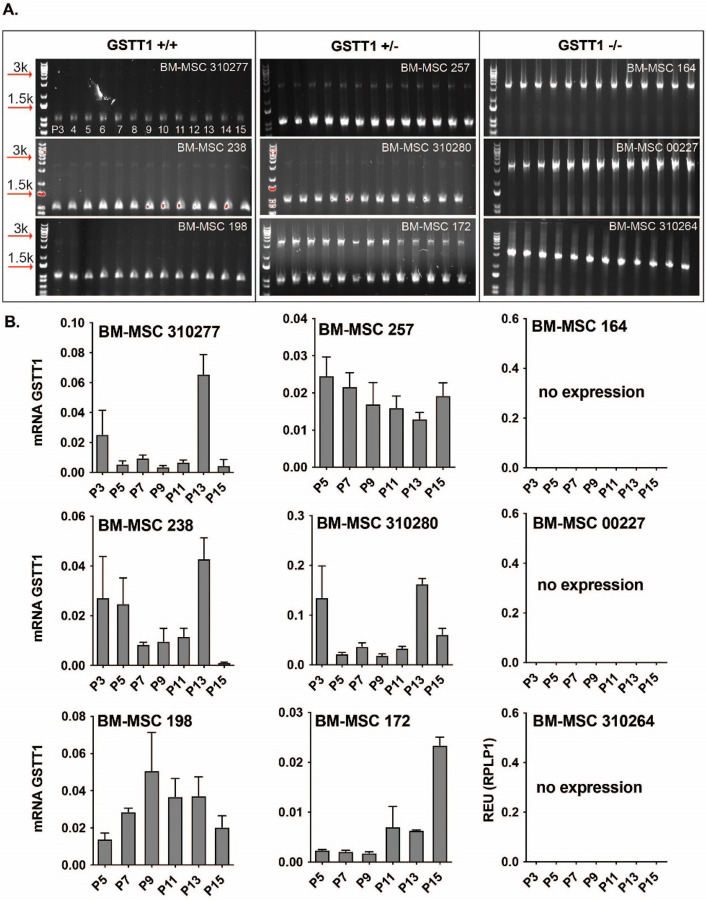
GSTT1 genotype of nine bone marrow-derived mesenchymal stem cell (BM-MSC) groups derived from different donors. **A)** Gel pictures of the amplified product obtained from multiplex PCR. BM-MSC groups 310277, 238, and 198 show a GSTT1 homozygous positive genotype, BM-MSC groups 257, 310280, and 172 show a GSTT1 heterozygous genotype, and BM-MSC groups 164, 00227, and 310264 show a GSTT1 homozygous negative genotype. **B)** Bar graphs depicting the average mRNA expression of the GSTT1 quantification using RT-qPCR. The relative gene expression is normalized to the respective RPLP0 expression.

BM-MSCs from multiple donors were genotyped to determine GSTT1 status using a multiplex PCR wherein the first primer pair amplified the GSTT1 gene which is approximately 1.5 kb long, whereas the second primer amplified the region flanking the GSTT1 gene, with an approximate size of 3.1 kb. The location in the genome of the forward and reverse primers used are shown in Fig. 1, **Panels A-B.** Genotyping allowed the grouping of BM-MSCs into three categories: GSTT1^+/+^ (homozygous positive), GSTT1^+/−^ (heterozygous) and GSTT1^−/−^ (homozygous null). BM-MSCs 310277,198, and 238 were identified to be GSTT1^+/+^; BM-MSCs 310280, 257, and 172 were observed to have a GSTT1^+/−^ genotype, and BM-MSCs 164, 310264, and 00227 were determined to be GSTT1^−/−^. The genotyping results are shown in Fig. 2, **Panel A**. Additionally, mRNA quantification showed that the homozygous null BM-MSCs showed no expression of GSTT1 mRNA whereas the heterozygous and homozygous positive groups showed positive expression of this gene as shown in Fig. 2, **Panel B.** Nevertheless, it was noted that GSTT1 gene expression was independent of passage number. While some BM-MSC groups showed higher expression in later passages (BM-MSC 172, BM-MSC 280, BM-MSC 238 and BM-MSC 310277), other donors showed higher expression in earlier passages (BM-MSC 257 and BM-MSC 198). No significant differences in GSTT1 gene expression were observed between the homozygous positive and heterozygous samples.

### 2. GSTT1 null genotype is predictive of higher proliferative potential in BM-MSCs

**Figure 3:**
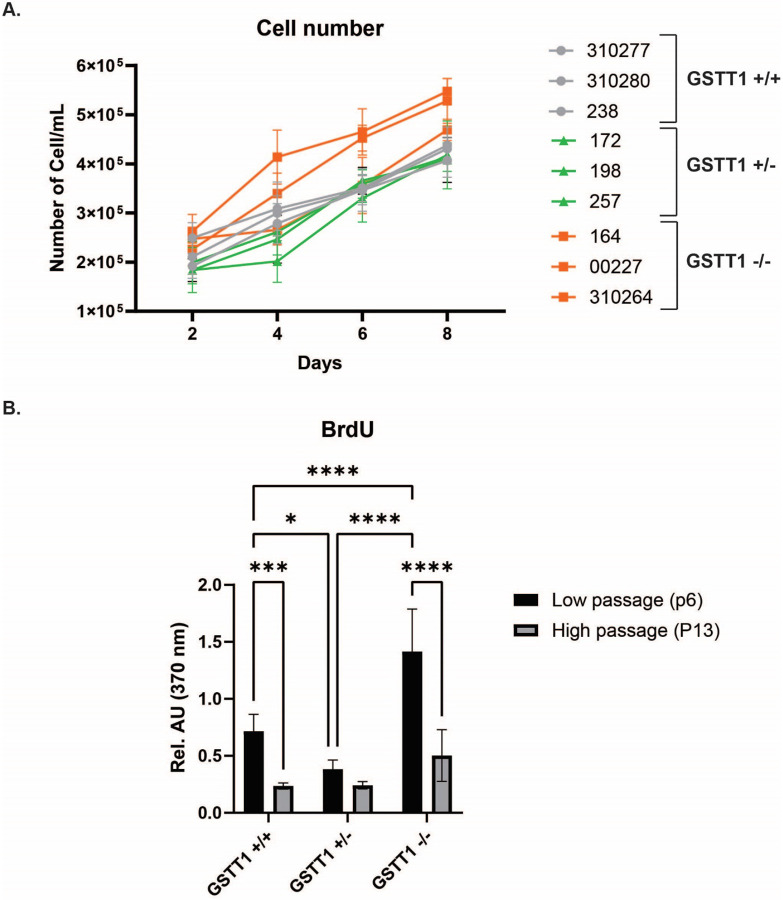
Bone marrow-derived mesenchymal stem cell (BM-MSC) proliferative potential. **A)** 8-day growth curve of nine BM-MSC groups divided into their GSTT1 genotypic groups. GSTT1 homozygous positive BM-MSCs are shown in gray dots, GSTT1 heterozygous BM-MSCs are shown in green triangles, and GSTT1 homozygous negative BM-MSCs are shown in orange squares. The average number of cells after nine cell counts is shown in the graph. Significance was achieved through an unpaired two-tail t-test. **B)** Bar graphs depicting the average absorbance readings at 370nm from the bromodeoxyuridine (BrdU) assay. Three readings for each group at low passages (P6) and high passages (P12) were performed and averaged. Data analysis was performed with one-way ANOVA.

The proliferative capacity of BM-MSCs was analyzed through a growth curve and a BrDU assay. From the 8-day growth curve, it was observed that BM-MSCs with a GSTT1 null genotype had a significantly higher proliferative potential when compared to the other two genotypes as shown in Fig. 3, **Panel A**. At day 8, the GSTT1 null groups had significantly more cells than BM-MSCs from the other two groups. This difference was observed mainly in BM-MSCs 164 and 00227. The compact letter display for significant differences is shown in Supplemental Table 1. Additionally, a BrdU assay was performed with one representative sample for each GSTT1 genotype at low passages (P6) and high passages (P12). Our results indicate that BM-MSCs with a GSTT1^−/−^ genotype showed significantly higher proliferation capacity compared to GSTT1^+/+^ and GSTT1^+/−^ BM-MSCs at both young passages (p-value < 0.0001) as shown in Fig. 3, **Panel B**.

### 3. BM-MSC telomere length attrition and hTERT expression are not related to GSTT1 genotype

**Figure 4:**
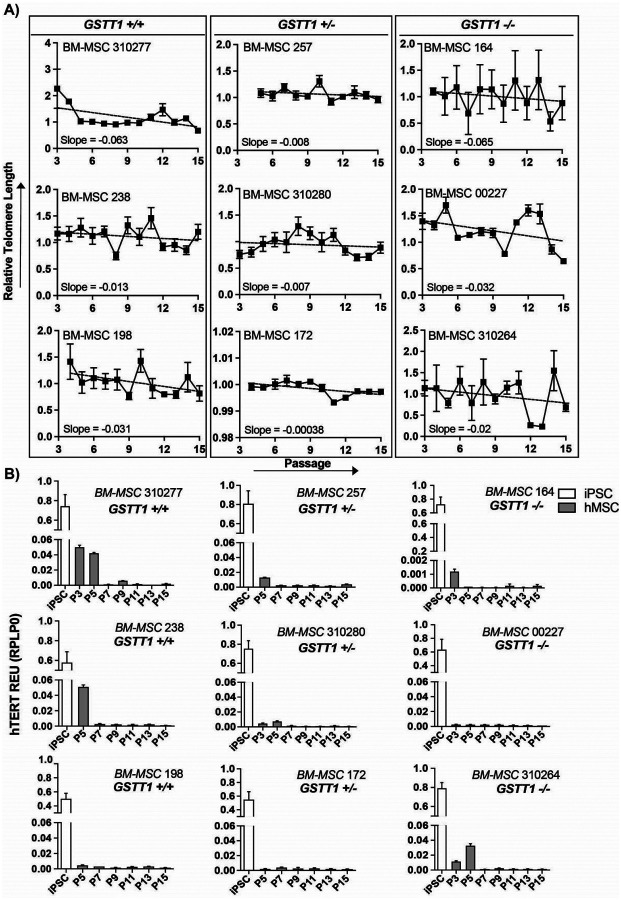
Telomere length and telomerase reverse transcriptase (hTERT) expression of bone marrow-derived mesenchymal stem cells (BM-MSCs) isolated from nine different donors. **A)** Relative telomere length at passages 3–15 from BM-MSCs. The slope of the best fit line (dotted line) is listed at the bottom of each graph. **B)** Bar graphs depicting the average hTERT relative expression normalized to RPLP0 measured by RT-qPCR. The white bar on the left of each graph depicts the hTERT expression of induced pluripotent stem cells (iPSCs). The gray bars represent hTERT expression of BM-MSCs from passage 3/5–15.

In order to assess BM-MSC replicative capacity, their telomere length was assessed from passage 3 to passage 15. The results show that all BM-MSC groups experienced telomere shortening as shown in Fig. 4, **Panel A**. Even though there was fluctuation in the recorded telomere lengths from passage to passage, the overall slope was negative in all BM-MSC groups. This shortening process did not appear to be related to the GSTT1 genotype but to be donor-dependent. From the tested groups, BM-MSC 172 showed the slowest shortening rate, with a slope of −0.00038 while BM-MSC 164 showed the fastest shortening, with a slope of −0.065.

Additionally, this study analyzed the expression of the hTERT gene. hTERT or human Telomere Retro Transcriptase is a gene associated with the synthesis of telomere lengthening. In general, normal diploid mature cells do not express the hTERT gene since this gene is usually expressed by pluripotent cells. The results showed that all BM-MSC groups had low hTERT gene expression with no significant differences between each other as shown in Fig. 4, **Panel B**. Nevertheless, hTERT expression is higher at lower passages for some BM-MSC groups and decreases as BM-MSCs are passaged. hTERT gene expression does not appear to be related to the GSTT1 genotype status.

### 4. GSTT1 genotype status affects the expression of p21 Waf1 and p14ARF but not the expression of p16INK4A, γH2AX and TP53

**Figure 5:**
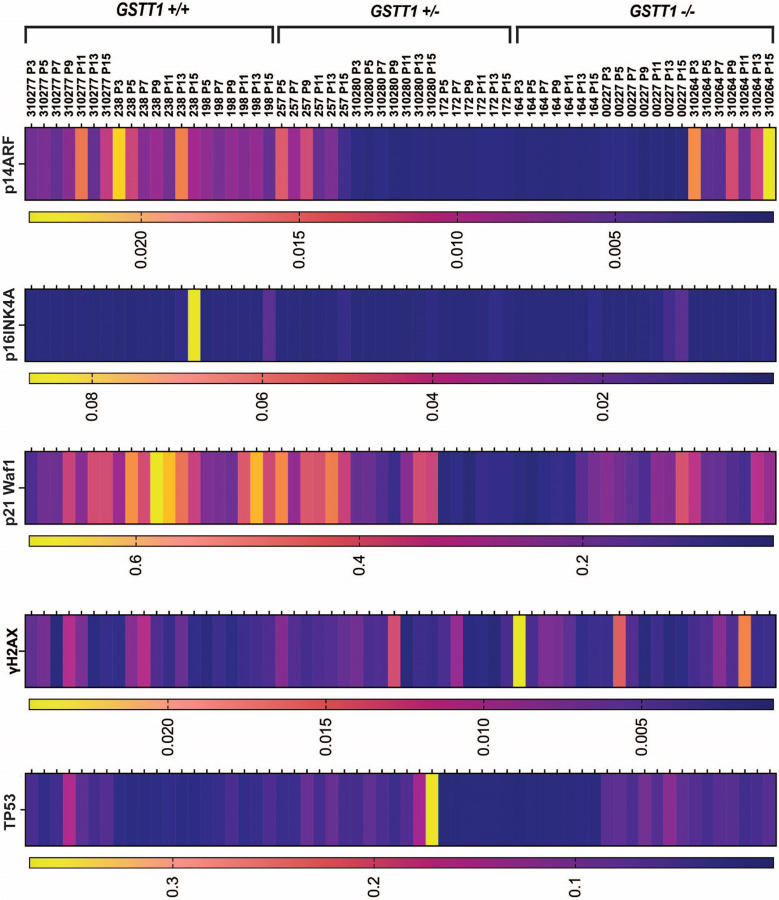
Gene expression of senescence-associated genes throughout long-term culture. p14ARF, p16INK4A, p21 Waf1, γH2AX, and TP53 were quantified at each passage in nine different bone-marrow derived mesenchymal stem cell (BM-MSCs) groups isolated from different donors. BM-MSCs are grouped by their genotype as GSTT1 homozygous positive (GSTT1 +/+), GSTT1 heterozygous (GSTT1 +/−), and GSTT1 homozygous negative (GSTT1 −/−). Gene expressions are normalized to their respective RPLP0 expressions. Data analysis was performed using a one-way ANOVA, n=3 for each group.

To assess the effects of GSTT1 expression in senescence-related genes, RT-qPCR was used to quantify p14ARF, p16INK4A, p21 Waf1, TP53 and γH2AX in GSTT1 homozygous positive, heterozygous, and homozygous negative BM-MSC groups as shown in Fig. 5. The results show that GSTT1 homozygous positive BM-MSCs 310277, 238, and 198 showed a fluctuating but high expression of p14ARF when compared to most of the heterozygous or homozygous negative samples. Two of the three GSTT1 heterozygous samples (310280 and 172) and homozygous negative samples (164 and 00227) had low expression of p14ARF even at high passages. However, GSTT1 heterozygous BM-MSC 257 and GSTT1 homozygous negative BM-MSC 310264 showed a fluctuating but relatively high expression of p14ARF at young passages. Furthermore, p16INK4A showed a mostly uniform and low expression at young passages regardless of the GSTT1 genotype. It increased with passages in most BM-MSC groups, seeing mostly non-significant increases at passages 13–15 except for the homozygous positive 238 group, in which p16INK4A expression increased significantly from P13-P15 (p-value < 0.0001).

Our results showed that p21 Waf1 expression increased with passage and showed the greatest but mostly non-significant differences between genotypic groups. Homozygous positive BM-MSC groups 238 and 198 showed the greatest p21 Waf1 expression at higher passages followed by homozygous positive 310277 and heterozygous 257 groups. Nevertheless, heterozygous 172 had the lowest p21 Waf1 expression even at high passages followed by homozygous negative 164. Then, γH2AX expression showed high variation between passages with no specific pattern or clear relation to genotype. TP53 showed the highest expression in heterozygous 310280 followed by homozygous negative 00227. Heterozygous 172 showed the lowest expression of TP53 followed by homozygous positive 238. However, a clear relationship between genotype and TP53 was not detected. The compact letter display that indicates significant differences between groups at each passage is shown in Supplemental Table 2.

### 5. GSTT1 genotype does not influence TWIST1/2 expression

**Figure 6:**
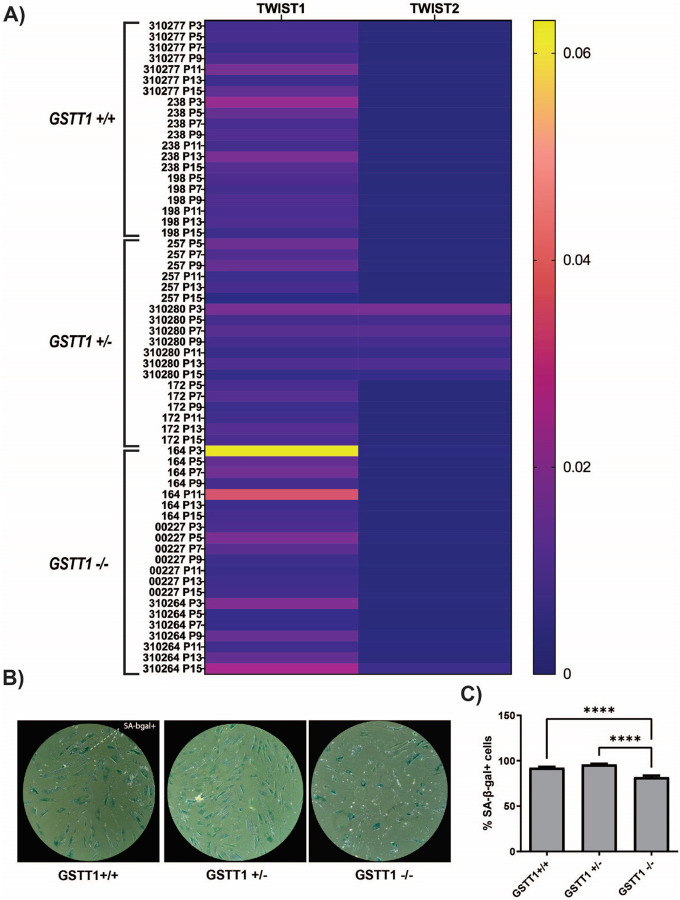
TWIST1/2 and senescence-associated β galactosidase (SA-β-gal) expression in bone marrow-derived mesenchymal stem cells (BM-MSCs) during and after expansion. **A)** TWIST1 and TWIST2 gene expression in BM-MSCs measured through RT-qPCR at multiple passages during long-term in vitro culture. BM-MSCs are grouped by their genotype as GSTT1 homozygous positive (GSTT1 +/+), GSTT1 heterozygous (GSTT1 +/−), and GSTT1 homozygous negative (GSTT1 −/−). **B)** SA-β-gal staining from three selected BM-MSC groups, one from each genotype. **C)** Bar graphs depicting the average number of SA-β-gal positive cells from each well divided by the total number of cells. Data analysis was performed using a one-way ANOVA, n=3 for each group.

In addition to the senescence-related genes quantified in this study, genes related to cell proliferation were analyzed, namely TWIST1 and TWIST2. The results from this study showed that TWIST1 expression varied at each passage with mostly non-significant differences between GSTT1 genotypes. Interestingly, homozygous negative 164 had the highest expression of TWIST1, particularly at passage 3 and 11. On the other hand, TWIST2 showed low expression in most samples except for the GSTT1 heterozygous BM-MSC 310280 which had significantly higher expression compared to the other samples as shown in Fig. 6, **Panel A**. The compact letter display that indicates significant differences between groups at each passage is shown in Supplemental Table 3.

### 6. GSTT1 null BM-MSCs have the lowest percentage of senescent cells after long-term in vitro culture.

To quantify the percentage of senescent cells, SA-β-gal staining was performed in cells after long-term in vitro culture (passage 15) in three representative BM-MSC groups (one from each genotype). SA-β-gal expression is known to be part of BM-MSC SASP and it is a reliable strategy to identify senescent cells. The results show that GSTT1 negative BM-MSCs have a significantly lower percentage of senescent cells in culture compared to homozygous positive (p-value < 0.0001) and heterozygous (p-value < 0.0001) BM-MSCs as shown in Fig. 6, **Panel B**. Moreover, no significant differences in SA-β-gal expression between the homozygous positive and heterozygous groups were observed.

### 7. GSTT1 genotype has a significant effect on the expression of senescence-associated genes at higher passages

In addition to the genes directly involved in cell cycle arrest and DNA damage repair, other genes known to be part of the senescence-associated secretory phenotype (SASP) or key BM-MSC processes were quantified. Here, the expressions of IL-6, SOD1, EZH2, ACTA2, and PDXN were measured at multiple passages to study the effect of long-term culture and GSTT1 genotype on their expression. IL-6 expression increases with increasing passage while EZH2 expression decreases with increasing passage in all three GSTT1 genotypes. However, the expression of both IL-6 and EZH2 is significantly higher in the homozygous positive BM-MSCs compared to the homozygous negative BM-MSCs when they reached higher passages. It is to be noted that, at low passages, the GSTT1 homozygous positive group had the lowest and highest expression of IL-6 and EZH2 respectively and experienced the greatest shifts in gene expression with long-term culture.

Then, SOD1 expression increases with passage in all BM-MSC groups. However, it significantly increases only in the GSTT1 homozygous positive group. Additionally, SOD1 expression at high passages in the GSTT1 homozygous positive group is significantly higher than its expression in the other two BM-MSC groups. The other two genes quantified in this study, namely ACTA2 and PDXN, do not show a clear trend of increasing or decreasing with passage, suggesting that the expression pattern is more heterogeneous across BM-MSC groups. Interestingly, ACTA2 expression at high passages was significantly higher in the GSTT1 negative group when compared to the other two BM-MSC groups. Moreover, PDXN expression was significantly higher in GSTT1 homozygous positive BM-MSCs when compared to the other BM-MSC groups at high passages.

### 8. BM-MSCs with a GSTT1 null genotype are resistant to some of the phenotypic changes produced by irradiation

Besides long-term in vitro culture, BM-MSC irradiation was performed as an additional aging model to assess the impact of GSTT1 genotype on BM-MSC senescence. Then, the percentage of senescent cells was assessed through SA-β-gal staining and the expression of senescence-associated genes 53BP1, γH2AX, p14ARF, p16INK4A, p21 Waf1, TP53, TWIST1, and TWIST2 was quantified.

**Figure 7:**
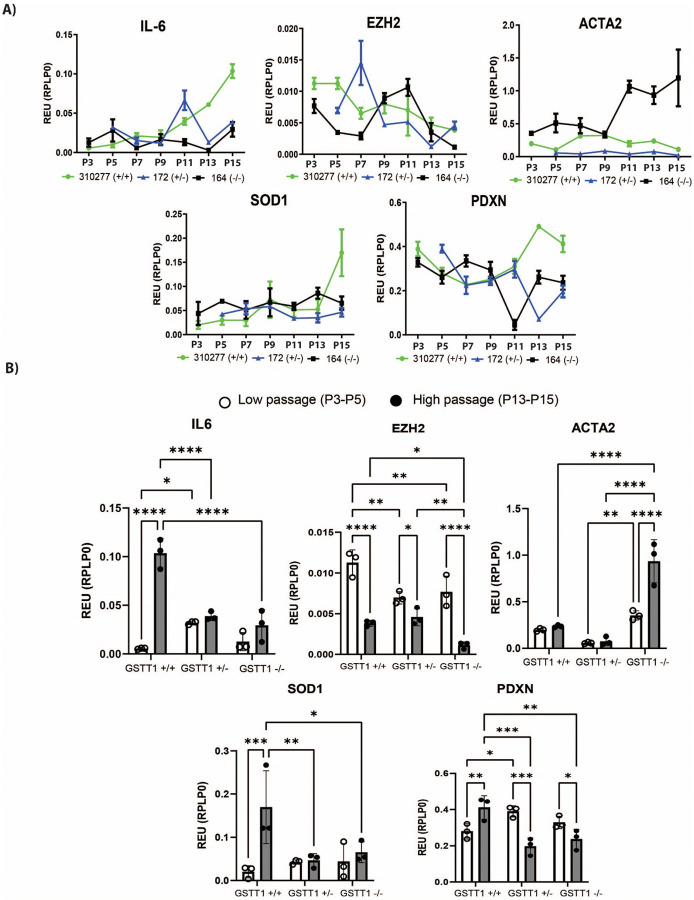
Gene expression of IL-6, EZH2, ACTA2, SOD1, and PDXN throughout long-term in vitro culture of bone marrow-derived mesenchymal stem cells (BM-MSCs) measured through RT-qPCR. Gene expression is normalized to the respective RPLP0 expression. **A)** Graphs depicting the average gene expression every two passages from passage 3/5 to passage 15 of three selected BM-MSC groups, one from each genotype. **B)** Bar graphs comparing gene expression averages at low passages (P3/P5) and high passages (P13/P15) and between genotypic groups. Data analysis was performed using a two-way ANOVA, n=3 for each group.

Our results show that, with irradiation, the percentage of senescent cells increases in all BM-MSC groups as shown in Fig. 7, **Panel A**. However, the percentage of senescent cells is significantly lower in the GSTT1 null group compared to the other two groups.

Additionally, gene expression results after irradiation show some interesting patterns as shown in Fig. 7, **Panel B.** The expressions of 53BP1 and p21 Waf1 significantly increase in the heterozygous and homozygous positive groups after irradiation. However, no significant changes for these genes were observed in the GSTT1 negative group, resulting in a significantly lower expression post-irradiation. Nevertheless, p16INK4A experienced a similar pattern but resulted in a significantly lower expression post-irradiation in the GSTT1 null group only when compared to the GSTT1 homozygous positive group. Moreover, γH2AX, p14ARF, and TP53 show similar expression patterns with irradiation no matter the genotypic group. Interestingly, although TWIST1 and TWIST2 expressions are initially higher in the GSTT1 negative group, they significantly decrease with irradiation. This phenomenon is only observed in the homozygous negative group as no significant changes are observed in the other two genotypic groups.

## DISCUSSION

In vitro expansion of BM-MSCs is necessary to obtain the necessary number of cells for clinical use [8]. However, extended culture often leads to cellular senescence, or a state of irreversible growth arrest while cells remain metabolically active [24, 25]. This phenomenon negatively affects BM-MSCs potency, leading to a diminished proliferative potential, decreased multipotentiality, and a modified secretome as part of their senescence-associated secretory phenotype (SASP)[9, 10, 26]. Notably, different groups have reported BM-MSCs populations with high proliferative potential and sustained phenotype despite prolonged culture [11, 27–29]. This can be partially attributed to the well-acknowledged heterogeneity that BM-MSCs intrinsically possess. However, this phenomenon is not well understood, leading to a lack of reliable strategies to identify BM-MSC populations that can undergo in vitro expansion without compromising their regenerative potential.

Previously, the GSTT1 gene, present in 14–30% of the population[16], has been studied in the context of BM-MSC proliferative potential. Sathiyanathan *et al*. showed that BM-MSCs lacking the GSTT1 gene can proliferate faster and possess longer telomeres than their GSTT1 positive counterparts [23]. Building upon these findings, we further assess how GSTT1 genotype affects BM-MSC phenotype during long-term in vitro culture and irradiation, discovering that a GSTT1 null genotype provides resistance to senescence and some of the changes associated with it. Consistent with earlier reports, we observed that cells with a GSTT1 null genotype proliferate faster than BM-MSCs with GSTT1 positive genotypes [23]. Furthermore, SA-β-gal expression, a well acknowledged marker of senescence [24], increased after subjecting BM-MSCs to both long-term culture and irradiation. Nevertheless, this increase was significantly attenuated in the GSTT1 null group when compared to the other two groups, implying a partial resistance to senescence.

Given the established link between telomere shortening and senescence [24, 26], we investigated BM-MSC telomere length and shortening rates. Our findings show no correlation between GSTT1 status and telomere length or rate of telomere shortening. Although Sathiyanathan *et al*. reported longer telomeres in GSTT1 null BM-MSCs, they similarly observed that the rate of telomere shortening was the same between the GSTT1 null and GSTT1 positive groups [23], which partially aligns with our results. Similarly, hTERT expression did not show any correlation to GSTT1 genotype, suggesting that neither the telomere length nor hTERT are responsible for the increased proliferative abilities and resistance to senescence in GSTT1 null BM-MSCs.

We then investigated whether GSTT1 status affects the expression of several genes related to senescence and cell cycle arrest in BM-MSCs, namely p14ARF, p16INK4A, p21 Waf1, γH2AX, 53BP1, and TP53. Both p16INK4A and p21 Waf1 have been studied in the context of senescence and have been shown to be elevated after long-term culture and following irradiation [26, 30–33]. A similar trend was observed in most of the BM-MSC groups during this study. However, the GSTT1 genotype had an important effect on the expression of these cyclin-dependent kinase inhibitors, indicating that a GSTT1 null genotype leads to a diminished increase in p16INK4A expression post-irradiation and p21 Waf1 expression during long-term culture and following irradiation. These results suggest that a GSTT1 null genotype can lower that percentage of cells undergoing cell cycle arrest, thus lowering the percentage of senescent cells in culture.

Similarly, p14ARF expression was generally higher in the GSTT1 homozygous positive BM-MSC groups even at earlier passages during expansion, except for the GSTT1 null 310264 and GSTT1 heterozygous 257, indicating that other variables beyond GSTT1 genotype may influence its regulation. Notably, p14ARF expression did not significantly differ between GSTT1 genotypes after irradiation. Even through p14ARF is not as thoroughly studied as p16INK4A and p21 Waf1 in the context of cell senescence, this cyclin-dependent kinase inhibitor has important p53-dependent and p53-independent functions, inhibiting p53 suppression and interacting with multiple proteins involved in chromosome stability and transcription [35, 36]. Interestingly, this study observed a decrease in p14ARF expression with passage in some BM-MSC groups, a trend that has been recorded previously [9] which might suggest other regulatory pathways for p14ARF expression. Although p16INK4A, p21 Waf1, and p14ARF are involved in the activation of p53 (involved in the conservation of the DNA integrity)[37], TP53, the gene responsible for coding this protein, did not seem to be related to GSTT1 genotype during long-term culture or irradiation.

To assess DNA damage repair responses, γH2AX and 53BP1 expressions were evaluated since these two proteins are involved specifically in double-strand breaks, a DNA lesion that can trigger permanent growth arrest and senescence [38]. γH2AX and 53BP1 are typically upregulated when BM-MSCs are exposed to irradiation [38–40]. As expected, γH2AX increased in all BM-MSC groups after irradiation with no genotype-specific differences, indicating that all BM-MSC genotypes can activate the γH2AX repair mechanism when DNA double-strand breaks are present, preserving the genomic integrity when cells are exposed irradiation. However, when γH2AX was quantified during prolonged expansion, a link between passage number or BM-MSC genotype and γH2AX expression was not found, consistent with previous reports showing impairment of the DNA damage response in BM-MSCs as they are extensively passaged [41, 42]. On the other hand, when 53BP1 was quantified after irradiation, its expression increased in all BM-MSC groups except for the GSTT1 null group. This is unexpected since 53BP1 is usually co-expressed with γH2AX post-irradiation [40, 43]. Nonetheless, this could mean that GSTT1 null BM-MSCs prefer the homologous recombination double-strand break repair mechanism, which do not necessarily require the 53BP1 protein, in contrast to the non-homozygous end joining pathway [38].

We further quantified the expression of TWIST1 and TWIST2, genes involved in maintaining BM-MSC stemness [44]. Our results indicate that, at low passages, TWIST1 expression was the highest in the GSTT1 null groups and decreased with long-term culture in all BM-MSC groups. Previously, TWIST1 expression in BM-MSCs has been correlated with a higher proliferative capacity and resistance to senescence [27, 45–47], agreeing with our results as shown in the growth curve and TWIST1 quantification. Interestingly, after irradiation, the GSTT1 null group was the only one that experienced a significant decrease in TWIST1 and TWIST2 expression, which might be due to them having the highest TWIST1 and TWIST2 expression pre-irradiation in the first place. Moreover, this decrease in TWIST1 and TWIST2 expression could suggest that a GSTT1 null BM-MSCs are not able to conserve an advantage in their proliferative capacities post-irradiation compared to the other BM-MSC genotypes, but more research on the topic is encouraged.

After that, genes usually altered with senescence, IL-6, SOD1, EZH2, ACTA2, and PDXN, were quantified to evaluate how SASP changes depending on the genotype of BM-MSCs. With long-term culture, there were changes in the secretome of BM-MSCs from all genotypes. However, the GSTT1 null group expressed lower levels of IL-6, a pro-inflammatory cytokine and part of BM-MSC SASP[10, 31, 48] and PDXN, whose elevated levels might contribute to oxidative stress and apoptosis[49], indicating that GSTT1 null BM-MSCs have a less inflammatory and pro-apoptotic secretome when compared to the other BM-MSC groups. Additionally, the GSTT1 null group showed signs of higher multipotentiality as shown by the elevated levels of ACTA2, a myogenic differentiation marker [50]. Finally, EZH2 levels decreased in all BM-MSC groups but were the lowest in the GSTT1 null group. Previously, EZH2 expression has been shown to diminish senescence and SASP in BM-MSCs [51, 52], therefore its decrease in expression with long-term passage is predicted. However, the particularly low expression of EZH2 in the GSTT1 null group is unexpected and needs to be further studied since it does not seem to increase the number of senescent cells as shown by the SA-β-gal secretion.

Overall, the absence of the GSTT1 gene in BM-MSC grants these cells with partial resistance to senescence, increasing their proliferative potential and decreasing some of their SASP. Even though a GSTT1 null genotype has been shown to be deleterious in the context of diseases like cancer [18, 20], it can have important advantages in regenerative medicine applications. This specific genotype can be used as a biomarker to screen for BM-MSC populations that will conserve their potency even after long-term in vitro culture. Consequently, this will enable cell manufacturing companies to produce high numbers of BM-MSCs through expansion without compromising their quality and potency, generating more consistent and effective results when applied in a clinical setting. Further research on GSTT1 genotypes and their implications in BM-MSC phenotype is encouraged.

## CONCLUSIONS

In conclusion, this study demonstrates that GSTT1 null BM-MSCs exhibit an enhanced proliferative capacity and partial resistance to senescence. Additionally, these cells have reduced SASP and expression of important senescence-associated genes, namely p21 Waf1, p14ARF, 53BP1, IL-6, and PDXN. Additionally, BM-MSCs with this genotype express TWIST1 and ACTA2 at higher levels, something that might contribute to their stemness and proliferation potential. While further investigations are needed, a GSTT1 null genotype can serve as a potential biomarker to identify BM-MSCs populations that can be expanded in vitro without compromising their quality too much.

## MATERIALS AND METHODS

### Genotyping

For genotyping, BM-MSC genomic DNA was isolated from 80% confluent T75 flasks using DNeasy Blood & Tissue Kit (Qiagen, cat.no. 69504) following manufacturer’s instructions. Briefly, cells were lysed using the lysis buffer with proteinase K. Then, lysates were transferred into a binding membrane and washed several times using guanidine/ethanol buffer. Finally, DNA was eluted using 200 μL of elution buffer and quantified using a NanoPhotometer^®^ N60 (IMPLEN). Subsequently, PCR was performed by multiplexing using Platinum Multiplex PCR Master Mix (Applied Biosystems, cat.no. 446429) consisting of 2 different sets of primers shown in Supplemental Table 4. This strategy was used to detect homozygous and heterozygous BM-MSC populations as described in Buchard *et al*. [53]. The first set of primers detects the deletion of GSTT1. When the gene is present, the DNA fragment between the forward and reverse primers will be greater than 60 kb, which is too long for amplification; however, if deletion is present (i.e. absence of GSTT1), the DNA fragment will be around 3.1 kb which corresponds to the pseudo GSTT1 gene and results in amplification. The second set of primers detects the presence of the GSTT1 gene, yielding a fragment of around 1.5 kb that will undergo amplification if the gene is present and resulting in no amplification if the gene is absent. The PCR profile used for this study includes heating a thermal cycler CFX96 (Bio-Rad) at 95°C for 5 min followed by a cycle of 95°C for 2 min, 60°C for 30 sec, and 72°C for 1 min for the last cycle, followed by an additional final extension at 72° c for 10 min. The amplicons obtained were run on a 0.8% agarose/TBE electrophoresis gel at 50V for 60 min, followed by analyses using Chemidoc (Bio-Rad) gel documentation system.

### Culture and expansion of mesenchymal stem cells

BM-MSCs from healthy male individual donors between the age of 20–30 were cultured in T75 flasks using low-glucose DMEM media supplemented with 1% antibiotic (penicillin-streptomycin), 1% of L-Glutamine, and 10% fetal bovine serum. The cells were cultured under standard conditions of 5% CO_2,_ and 37°C in sterile incubators and continuously expanded from passage 4–5 until passage 15. For expansion, BM-MSCs were harvested using TrypLE^™^ (Thermo Fisher Scientific, cat.no. 12605010) once they reached 80% confluency. Then, they were reseeded at a density of 5,000 cells/cm^2^ into new T75 flasks. At each passage, a fraction of cells was collected for DNA and RNA analysis.

### Irradiation of BM-MSCs

BM-MSCs from each genotype were seeded at a density of 5,000 cells/cm^2^ in 6-well plates and allowed to reach 75% confluency. Prior to irradiation, cells were treated with fresh maintenance media. BM-MSCs were irradiated using an X-ray source for 5 minutes every day for three days to provide a total of 10 Gy dose strength. Post-irradiation, the cells were returned to normal culture conditions with no media change for the next 3 days. At 72 hours post-irradiation, cells were harvested for mRNA analysis to assay gene expression of senescence-related markers or fixed for assessment of senescence-associated beta-galactosidase (SA-β-gal) activity.

### Cell proliferation assay by bromodeoxyuridine incorporation

Three representative samples, one from each genotype group (BM-MSC 164 GSTT1^−/−^, BM-MSC 310277 GSTT1^+/+^, and BM-MSC 257 GSTT1^+/−^) were chosen to perform a bromodeoxyuridine (BrDU) assay. BM-MSCs at low passages (< P6) or high passages (P12<) were seeded at a density of 2000cells/well into 96-well plates with 6 replicates per sample. BrDU was performed using a BrDU ELISA Colorimetric kit (Roche, cat.no. 11647229001) following manufacturer’s instructions. Briefly, cells were incubated at 37°C with 5% CO^2^ for 24 hours. Then, BM-MSCs were checked for confluency and treated with S-phase DNA-specific primary antibody followed by an additional incubation for 24 hours. Plates were then heated for 1 hour at 60° C using a hot plate. After that, 200 μL of FixDenat solution was added to each well followed by a 30-minute incubation at ambient temperature. Then, the FixDenat solution was removed, and a secondary antibody was added. Next, cells were incubated for 90 min at ambient temperature. After removing the secondary antibody solution, cells were washed three times with 200 μL of 1X PBS. Finally, chromogenic substrate was added and incubated at room temperature for 25 minutes followed by measurement of the absorbance at 370 nm for 25, 30, and 35 minutes of incubation corresponding to BrDU activity.

### Real-time quantitative polymerase chain reaction

Total RNA from individual cell cultures were isolated using RNeasy Miniprep kit (Qiagen, cat.no. 74104) following manufacturer’s protocol recommendations. Briefly, BM-MSCs were lysed using a lysis buffer. Then, lysates were transferred into silica binding membrane and washed several times using wash buffers provided in the kit. RNA was then eluted using DNase/RNase free water followed by quantification and purity assessment using a NanoPhotometer^®^ N60 (IMPLEN). cDNA synthesis was performed using 500 ng mRNA using SuperScript^™^ VILO^™^ cDNA Synthesis Kit (Invitrogen, cat.no. 11754250) following manufacturer’s protocol. The cDNA was diluted to 50 ng/μL and approximately 5ng of cDNA was used as a template for all downstream real-time quantitative polymerase chain reaction (RT-qPCR) assays using SYBR Green. Primer sequences for all genes assayed in the study are listed in Supplemental Table 5. RT-qPCR was performed in a CFX96 (Bio-Rad) thermal cycler using standard reaction set up that comprised an activation at 95° C for 2 min, 40 cycles of 95°C for 1 min and 60°C for 30s of annealing and extension followed by a melt curve. All Ct values obtained were normalized to a housekeeping gene (RPLP0) and presented as a relative expression (2^–ΔΔCt^) as described previously [54].

### Telomere length analysis

The relative telomere lengths of BM-MSCs from multiple donors were quantified RT-qPCR using previously established protocols [54]. Genomic DNA of BM-MSCs at multiple passages was isolated using the DNeasy Blood & Tissue Kit (Qiagen, cat.no. 69504) following manufacturer’s instructions and used in a SYBR green-based RT-qPCR reaction with Tel 1 and Tel 2 primers and single copy genes of 36B4u and 36B4d. The primer sequences are shown in Supplemental Table 6. The thermal cycler CFX96 (BioRad) set up involved activation at 95°C for 1 min, followed by 40 cycles at 95°C (1min), 60°C (30 s), and 2 min at 54°C. Then, a melt curve analysis was performed. A modification in the reaction set up was applied for the single copy gene analyses which involve 40 cycles of the program: 1 min at 95°C, 30 sec at 60°C, and 1 min at 58°C. Standard curves for both telomere and single copy genes were first obtained from iPSC gDNA for the standard curve at the following dilutions: 100, 50, 25, 12.5, 6.25, 3.125, and 1.5625 ng/ml. Telomere length RT-qPCR using *Tel* primers and *36B4* primers was performed using 12.5 ng of gDNA (for each sample at specified passage in three replicates) and the data was analyzed using 2^–ΔΔCt^ as described previously [54].

### hTERT gene expression analysis

The expression of telomerase reverse transcriptase (hTERT), the gene responsible for telomere repeats, was assessed in all BM-MSC groups using RT-qPCR. As an internal control, mRNA from iPSCs was used since these cells have a robust expression of hTERT. The primer sequences for the *hTERT* gene are provided in Supplemental Table 6. RNA from BM-MSCs and iPSCs was isolated using RNeasy mini kit (Qiagen, cat.no. 74104) followed by quantification and reverse transcription as described in previous sections. RT-qPCR was performed using our established standard protocol (95°C for 2 min, 40 cycles of 95°C for 1 min and 60°C for 30 sec) in an CFX96 (BioRad) thermal cycler, followed by melt curve analysis. Data was analyzed using 2^–ΔΔCt^ as described previously [54] using RPLP0 as the housekeeping gene.

### Senescence-associated Beta-galactosidase (SA-b-gal) Staining

Representative BM-MSCs from each GSTT1 genotype (BM-MSC 164^−/−^, BM-MSC 310277^+/+^, and BM-MSC 257^+/−^) were cultured according to standard conditions and sampled at a specific passage/timepoint during expansion. Cells were seeded in glass bottomed dishes (MatTek, cat.no. P35GC-1.5–14-C) in maintenance media. At 75% confluency, cells were either irradiated for a total dose of 10Gy or were left non-irradiated in preparation for assessing SA-β-gal activity using the senescence-associated b-galactosidase staining kit (Cell Signaling, cat.no. 9860) following manufacturer’s instructions. Briefly, cells were fixed in 1X fixative solution for 15 min at room temperature followed by washes with 1X PBS. Fixed cells were stained with β-galactosidase Staining Solution. Dishes were sealed in parafilm and incubated at 37°C overnight in a dry incubator (no CO_2_). Post-incubation, cells were evaluated under the microscope for development of blue color which indicates SA-β-gal activity. In addition to the irradiation studies, SA-b-gal activity was assessed in continuously expanded cells until passage 15 to assess in vitro replicative senescence. Stained images were examined under bright field microscopy. Cells stained positively were counted to estimate the percentage of *SA-b-gal*^+^ cells.

### Statistical analysis

To compare phenotypic measurements and gene expression between the three genotypes, one-way ANOVA, two-way ANOVA, and *t*-test analyses where applicable were performed. An α value of 0.05 was used to determine significance in the compact letter display. For the bar graphs, * was used for a p-value ≤ 0.05, ** for a p-value ≤ 0.01, *** for a p-value ≤ 0.001, and **** for a p-value ≤ 0.0001.

## Supplementary Material

This is a list of supplementary files associated with this preprint. Click to download.

- Supplementaltables.pdf

## Figures and Tables

**Figure 8:**
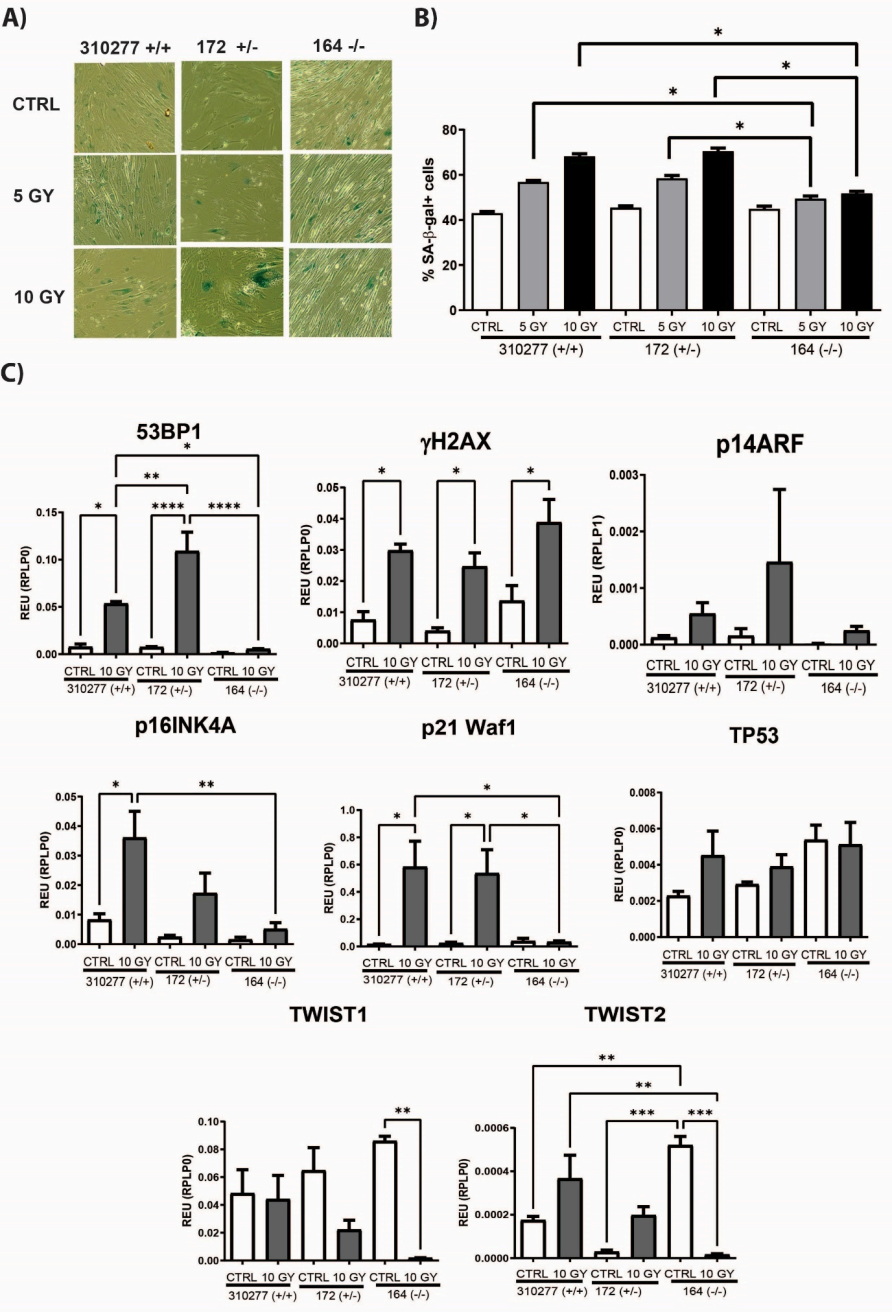
Senescence-associated β galactosidase (SA-β-gal) staining and senescence-associated gene expression after irradiation of bone marrow-derived mesenchymal stem cells (BM-MSCs).**A)** SA-β-gal expression of BM-MSCs without irradiation (CTRL) and after experiencing 5 and 10 GY of irradiation from three selected BM-MSC groups, one from each genotype. **B)** Bar graphs depicting the average number of SA-β-gal positive cells over the total number of cells. **C)** Bar graphs depicting the average gene expression of 53BP1, γH2AX, p14ARF, p16INK4A, p21 Waf1, TP53, TWIST1, and TWIST2 without irradiation (CTRL) and after 10 GY of irradiation exposure. Gene expressions were normalized to their respective RPLP0 expressions. Data analysis was performed using a one-way ANOVA, n=3 for each group.

## Data Availability

The datasets used and/or analyzed during the current study are available from the corresponding author on reasonable request

## References

[1] Maldonado V. V. , “Clinical utility of mesenchymal stem/stromal cells in regenerative medicine and cellular therapy,” Journal of Biological Engineering, vol. 17, no. 1, 2023, doi: 10.1186/s13036-023-00361-9. PMC10334654 37434264

[2] Samsonraj R. M., Raghunath M., Nurcombe V., Hui J. H., van Wijnen A. J., and Cool S. M., “Concise Review: Multifaceted Characterization of Human Mesenchymal Stem Cells for Use in Regenerative Medicine,” Stem cells translational medicine, vol. 6, no. 12, pp. 2173–2185, 2017, doi: 10.1002/sctm.17-0129. 29076267 PMC5702523

[3] Williams T. , “Versatility of mesenchymal stem cell-derived extracellular vesicles in tissue repair and regenerative applications,” Biochimie, vol. 207, pp. 33–48, 2023, doi: 10.1016/j.biochi.2022.11.011. 36427681

[4] Kabat M., Bobkov I., Kumar S., and Grumet M., “Trends in mesenchymal stem cell clinical trials 2004–2018: Is efficacy optimal in a narrow dose range?,” Stem cells translational medicine, vol. 9, no. 1, pp. 17–27, 2020, doi: 10.1002/sctm.19-0202. 31804767 PMC6954709

[5] Lozano-Rivas N. , “AB1011 Clinical trial of intravenous infusion of fucosylated bone marrow mesenchymal stem cells in patients with osteoporosis,” Annals of the rheumatic diseases, vol. 77, no. Suppl 2, p. 1625, 2018, doi: 10.1136/annrheumdis-2018-eular.4728.

[6] Theodosaki A. M. , “Bone Regeneration with Mesenchymal Stem Cells in Scaffolds: Systematic Review of Human Clinical Trials,” Stem cell reviews and reports, vol. 20, no. 4, pp. 938–966, 2024, doi: 10.1007/s12015-024-10696-5. 38407793 PMC11087324

[7] Li Y. , “Current status of clinical trials assessing mesenchymal stem cell therapy for graft versus host disease: a systematic review,” Stem cell research & therapy, vol. 13, no. 1, pp. 93-93, 2022, doi: 10.1186/s13287-022-02751-0. 35246235 PMC8895864

[8] Binato R. , “Stability of human mesenchymal stem cells during in vitro culture: considerations for cell therapy,” Cell proliferation, vol. 46, no. 1, pp. 10–22, 2013, doi: 10.1111/cpr.12002. 23163975 PMC6496525

[9] Maldonado V. V., Jensen H., Barnes C. L., and Samsonraj R. M., “Phenotypic changes associated with continuous long term in vitro expansion of bone marrow-derived mesenchymal stem cells,” Biochimie, vol. 234, pp. 62–75, 2025, doi: 10.1016/j.biochi.2025.04.002. 40209891 PMC13283746

[10] Samsonraj R. M., Law S. F., Chandra A., and Pignolo R. J., “An unbiased proteomics approach to identify the senescence-associated secretory phenotype of human bone marrow-derived mesenchymal stem cells,” Bone Reports, vol. 18, pp. 101674–101674, 2023, doi: 10.1016/j.bonr.2023.101674. 36994454 PMC10041468

[11] Heinrichs D. P., Maldonado V. V., Ardana I. K. K., Porter R. M., and Samsonraj R. M., “Assessing the Effects of Dasatinib on Mesenchymal Stem/Stromal Cells: Assessing the Effects of Dasatinib,” Cellular and molecular bioengineering, vol. 17, no. 6, pp. 609–618, 2024, doi: 10.1007/s12195-024-00830-1. 39926377 PMC11799476

[12] Needy T. , “Examining the Effects of Quercetin on Phenotypic Characteristics of Human Mesenchymal Stem Cells,” Cellular and molecular bioengineering, 2025, doi: 10.1007/s12195-025-00849-y. PMC12436255 40963712

[13] Wang T., Zhang J., Liao J., Zhang F., and Zhou G., “Donor genetic backgrounds contribute to the functional heterogeneity of stem cells and clinical outcomes,” Stem cells translational medicine, vol. 9, no. 12, pp. 1495–1499, 2020, doi: 10.1002/sctm.20-0155. 32830917 PMC7695629

[14] Maged G., Abdelsamed M. A., Wang H., and Lotfy A., “The potency of mesenchymal stem/stromal cells: does donor sex matter?,” Stem cell research & therapy, vol. 15, no. 1, pp. 112-112, 2024, doi: 10.1186/s13287-024-03722-3. 38644508 PMC11034072

[15] Turlo A. J. , “Mesenchymal Stromal Cell Secretome Is Affected by Tissue Source and Donor Age,” Stem cells (Dayton, Ohio), vol. 41, no. 11, pp. 1047–1059, 2023, doi: 10.1093/stmcls/sxad060. 37591507 PMC10631804

[16] Nakanishi G. , “GSTM1 and GSTT1 polymorphisms in healthy volunteers - a worldwide systematic review,” Drug metabolism reviews, vol. 54, no. 1, pp. 37–45, 2022, doi: 10.1080/03602532.2022.2036996. 35103568

[17] Guedi A. B. , “Glutathione S-transferase polymorphisms (GSTM1/GSTT1) outcomes in clinical profile and treatment responsiveness among Tunisian cohort of Parkinson’s disease,” JOURNAL OF NEURAL TRANSMISSION, vol. 132, no. 1, pp. 117–127, 2025, doi: 10.1007/s00702-024-02815-w. 39123072

[18] Ye Z. and Song H., “Glutathione s-transferase polymorphisms (GSTM1, GSTP1 and GSTT1) and the risk of acute leukaemia: A systematic review and meta-analysis,” European journal of cancer (1990), vol. 41, no. 7, pp. 980–989, 2005, doi: 10.1016/j.ejca.2005.01.014. 15862746

[19] Abbas M. , “Association of GSTM1 and GSTT1 gene polymorphisms with COVID-19 susceptibility and its outcome,” Journal of Medical Virology, vol. 93, no. 9, pp. 5446–5451, 2021, doi: 10.1002/jmv.27076. 33990973 PMC8242761

[20] Zhang W.-P., Yang C., Xu L.-J., Wang W., Song L., and He X.-F., “Individual and combined effects of GSTM1, GSTT1, and GSTP1 polymorphisms on lung cancer risk: A meta-analysis and re-analysis of systematic meta-analyses,” Medicine (Baltimore), vol. 100, no. 26, pp. e26104–e26104, 2021, doi: 10.1097/MD.0000000000026104. 34190143 PMC8257913

[21] Dasari S., Ganjayi M. S., and Meriga B., “Glutathione S-transferase is a good biomarker in acrylamide induced neurotoxicity and genotoxicity,” Interdisciplinary toxicology, vol. 11, no. 2, pp. 115–121, 2018, doi: 10.2478/intox-2018-0007. 31719782 PMC6829684

[22] Kudlova N., De Sanctis J. B., and Hajduch M., “Cellular Senescence: Molecular Targets, Biomarkers, and Senolytic Drugs,” International journal of molecular sciences, vol. 23, no. 8, p. 4168, 2022, doi: 10.3390/ijms23084168. 35456986 PMC9028163

[23] Sathiyanathan P. , “A genomic biomarker that identifies human bone marrow-derived mesenchymal stem cells with high scalability,” Stem Cells, vol. 38, no. 9, pp. 1124–1136, 2020, doi: 10.1002/stem.3203. 32510174

[24] Huang W., Hickson L. J., Eirin A., Kirkland J. L., and Lerman L. O., “Cellular senescence: the good, the bad and the unknown,” Nature reviews. Nephrology, vol. 18, no. 10, pp. 611–627, 2022, doi: 10.1038/s41581-022-00601-z. 35922662 PMC9362342

[25] Wagner K.-D. and Wagner N., “The Senescence Markers p16INK4A, p14ARF/p19ARF, and p21 in Organ Development and Homeostasis,” Cells (Basel, Switzerland), vol. 11, no. 12, p. 1966, 2022, doi: 10.3390/cells11121966. PMC9221567 35741095

[26] Liu J., Ding Y., Liu Z., and Liang X., “Senescence in Mesenchymal Stem Cells: Functional Alterations, Molecular Mechanisms, and Rejuvenation Strategies,” Frontiers in cell and developmental biology, vol. 8, pp. 258-258, 2020, doi: 10.3389/fcell.2020.00258. 32478063 PMC7232554

[27] Samsonraj R. M. , “Establishing Criteria for Human Mesenchymal Stem Cell Potency,” Stem cells (Dayton, Ohio), vol. 33, no. 6, pp. 1878–1891, 2015, doi: 10.1002/stem.1982. 25752682 PMC5363381

[28] DiGirolamo C. M., Stokes D., Colter D., Phinney D. G., Class R., and Prockop D. J., “Propagation and senescence of human marrow stromal cells in culture: a simple colony-forming assay identifies samples with the greatest potential to propagate and differentiate,” British journal of haematology, vol. 107, no. 2, pp. 275–281, 1999, doi: 10.1046/j.1365-2141.1999.01715.x. 10583212

[29] Muraglia A., Cancedda R., and Quarto R., “Clonal mesenchymal progenitors from human bone marrow differentiate in vitro according to a hierarchical model,” Journal of cell science, vol. 113, no. 7, pp. 1161–1166, 2000, doi: 10.1242/jcs.113.7.1161. 10704367

[30] Ho J. H., Chen Y.-F., Ma W.-H., Tseng T.-C., Chen M.-H., and Lee O. K., “Cell Contact Accelerates Replicative Senescence of Human Mesenchymal Stem Cells Independent of Telomere Shortening and p53 Activation: Roles of Ras and Oxidative Stress,” Cell transplantation, vol. 20, no. 8, pp. 1209–1220, 2011, doi: 10.3727/096368910X546562. 21176396

[31] Marote A. , “202 - Mesenchymal Stem/Stromal Cells: CELLULAR AGING SECRETES: A COMPARISON OF BONE MARROW DERIVED AND INDUCED MESENCHYMAL STEM CELLS AND THEIR SECRETOME OVER LONG TERM CULTURE,” Cytotherapy (Oxford, England), vol. 25, no. 6, pp. S86–S87, 2023, doi: 10.1016/S1465-3249(23)00286-4. 36152233

[32] Bai J., Wang Y., Wang J., Zhai J., He F., and Zhu G., “Irradiation-induced senescence of bone marrow mesenchymal stem cells aggravates osteogenic differentiation dysfunction via paracrine signaling,” American Journal of Physiology: Cell Physiology, vol. 318, no. 5, pp. C1005–C1017, 2020, doi: 10.1152/ajpcell.00520.2019. 32233952

[33] Fekete N. , “Effect of High-Dose Irradiation on Human Bone-Marrow-Derived Mesenchymal Stromal Cells,” Tissue engineering. Part C, Methods, vol. 21, no. 2, pp. 112–122, 2015, doi: 10.1089/ten.tec.2013.0766. 24918644 PMC4313408

[34] Yan J. , “The role of p21 in cellular senescence and aging-related diseases,” Molecules and cells, vol. 47, no. 11, p. 100113, 2024, doi: 10.1016/j.mocell.2024.100113. 39304134 PMC11564947

[35] Cilluffo D., Barra V., and Di Leonardo A., “P14ARF: The Absence that Makes the Difference,” Genes, vol. 11, no. 7, p. 824, 2020, doi: 10.3390/genes11070824. 32698529 PMC7397060

[36] Sherr C. J., “Divorcing ARF and p53: an unsettled case,” Nature reviews. Cancer, vol. 6, no. 9, pp. 663–673, 2006, doi: 10.1038/nrc1954. 16915296

[37] Borrero L. J. H. and El-Deiry W. S., “Tumor suppressor p53: Biology, signaling pathways, and therapeutic targeting,” Biochimica et biophysica acta. Reviews on cancer, vol. 1876, no. 1, pp. 188556–188556, 2021, doi: 10.1016/j.bbcan.2021.188556. 33932560 PMC8730328

[38] Panier S. and Boulton S. J., “Double-strand break repair: 53BP1 comes into focus,” Nature reviews.Molecular cell biology, vol. 15, no. 1, pp. 7–18, 2014, doi: 10.1038/nrm3719. 24326623

[39] Dickey J. S., Redon C. E., Nakamura A. J., Baird B. J., Sedelnikova O. A., and Bonner W. M., “H2AX: functional roles and potential applications,” Chromosoma, vol. 118, no. 6, pp. 683–692, 2009, doi: 10.1007/s00412-009-0234-4. 19707781 PMC3094848

[40] Tsvetkova A. , “γH2AX, 53BP1 and Rad51 protein foci changes in mesenchymal stem cells during prolonged X-ray irradiation,” Oncotarget, vol. 8, no. 38, pp. 64317–64329, 2017, doi: 10.18632/oncotarget.19203. 28969073 PMC5610005

[41] Bao X. , “Extended in vitro culture of primary human mesenchymal stem cells downregulates Brca1-related genes and impairs DNA double-strand break recognition,” FEBS open bio, vol. 10, no. 7, pp. 1238–1250, 2020, doi: 10.1002/2211-5463.12867. PMC7327915 32333827

[42] Hladik D. , “Long-term culture of mesenchymal stem cells impairs ATM-dependent recognition of DNA breaks and increases genetic instability,” Stem cell research & therapy, vol. 10, no. 1, pp. 218-218, 2019, doi: 10.1186/s13287-019-1334-6. 31358047 PMC6664790

[43] Marková E., Schultz N., and Belyaev I. Y., “Kinetics and dose-response of residual 53BP1 γ-H2AX foci: Co-localization, relationship with DSB repair and clonogenic survival,” International journal of radiation biology, vol. 83, no. 5, pp. 319–329, 2007, doi: 10.1080/09553000601170469. 17457757

[44] Franco H. L., Casasnovas J., Rodríguez-Medina J. R., and Cadilla C. L., “Redundant or separate entities?--roles of Twist1 and Twist2 as molecular switches during gene transcription,” Nucleic acids research, vol. 39, no. 4, pp. 1177–1186, 2011, doi: 10.1093/nar/gkq890. 20935057 PMC3045590

[45] Toriumi K. , “LRRC15 expression indicates high level of stemness regulated by TWIST1 in mesenchymal stem cells,” iScience, vol. 26, no. 7, pp. 106946–106946, 2023, doi: 10.1016/j.isci.2023.106946. 37534184 PMC10391581

[46] Voskamp C. , “TWIST1 CONTROLS CELLULAR SENESCENCE AND ENERGY METABOLISM IN MESENCHYMAL STEM CELLS,” European Cells and Materials, vol. 42, pp. 401–414, 2021, doi: 10.22203/eCM.v042a25. 34825700

[47] Voskamp C., Anderson L. A., Koevoet W. J., van Osch G. J., and Narcisi R., “Twist1 regulates cellular senescence in mesenchymal stem cells,” Osteoarthritis and cartilage, vol. 28, pp. S509–S510, 2020, doi: 10.1016/j.joca.2020.02.800.

[48] Zheng Y., Wu S., Ke H., Peng S., and Hu C., “Secretion of IL-6 and IL-8 in the senescence of bone marrow mesenchymal stem cells is regulated by autophagy via FoxO3a,” Experimental gerontology, vol. 172, pp. 112062–112062, 2023, doi: 10.1016/j.exger.2022.112062. 36526098

[49] Cheng G. and Shi R., “Mammalian peroxidasin (PXDN): From physiology to pathology,” Free radical biology & medicine, vol. 182, pp. 100–107, 2022, doi: 10.1016/j.freeradbiomed.2022.02.026. 35219848 PMC8957557

[50] Mistriotis P. , “NANOG Reverses the Myogenic Differentiation Potential of Senescent Stem Cells by Restoring ACTIN Filamentous Organization and SRF-Dependent Gene Expression,” Stem cells (Dayton, Ohio), vol. 35, no. 1, pp. 207–221, 2017, doi: 10.1002/stem.2452. 27350449

[51] Yang R. , “1,25-Dihydroxyvitamin D protects against age related osteoporosis by a novel VDR-Ezh2-p16 signal axis,” Aging cell, vol. 19, no. 2, pp. e13095-n/a, 2020, doi: 10.1111/acel.13095. 31880094 PMC6996957

[52] Wang T., Chen J., Qu B., Zhou D., and Hong Z., “Scutellarin Alleviates Bone Marrow Mesenchymal Stromal Cellular Senescence via the Ezh2-Nrf2 Signalling Axis in Diabetes-Induced Bone Loss,” Cell proliferation, vol. 58, no. 4, pp. e13790-n/a, 2025, doi: 10.1111/cpr.13790. 39668494 PMC11969241

[53] Buchard A., Sanchez J. J., Dalhoff K., and Morling N., “Multiplex PCR Detection of GSTM1, GSTT1, and GSTP1 Gene Variants: Simultaneously Detecting GSTM1 and GSTT1 Gene Copy Number and the Allelic Status of the GSTP1 Ile105Val Genetic Variant,” The Journal of molecular diagnostics : JMD, vol. 9, no. 5, pp. 612–617, 2007, doi: 10.2353/jmoldx.2007.070030. 17916600 PMC2049057

[54] Samsonraj R. M., Raghunath M., Hui J. H., Ling L., Nurcombe V., and Cool S. M., “Telomere length analysis of human mesenchymal stem cells by quantitative PCR,” Gene, vol. 519, no. 2, pp. 348–355, 2013, doi: 10.1016/j.gene.2013.01.039. 23380569

